# Important periods of weight development in childhood: a population-based longitudinal study

**DOI:** 10.1186/1471-2458-14-160

**Published:** 2014-02-13

**Authors:** Kari Glavin, Mathieu Roelants, Bjørn Heine Strand, Pétur B Júlíusson, Kari Kveim Lie, Sølvi Helseth, Ragnhild Hovengen

**Affiliations:** 1 Oslo and Akershus University College of Applied Sciences, (Nursing), P.O. Box 4, St.Olav plass, 0130 Oslo, Norway; 2 Department of Public Health and Primary Care, KU Leuven – University of Leuven, Leuven, Belgium; 3 Division of Epidemiology, Norwegian Institute of Public Health, P.O. Box 4404, Nydalen, 0403 Oslo, Norway; 4 Department of Clinical Science, Section of Paediatrics, University of Bergen, Bergen, Norway

**Keywords:** Child, Overweight, Obesity, BMI

## Abstract

**Background:**

Identifying important ages for the development of overweight is essential for optimizing preventive efforts. The purpose of the study was to explore early growth characteristics in children who become overweight or obese at the age of 8 years to identify important ages for the onset of overweight and obesity.

**Methods:**

Data from the Norwegian Child Growth Study in 2010 (N = 3172) were linked with repeated measurements from health records beginning at birth. Weight and height were used to derive the body mass index (BMI) in kg/m^2^. The BMI standard deviation score (SDS) for each participant was estimated at specific target ages, using a piecewise linear mixed effect model.

**Results:**

At 8 years of age, 20.4% of the children were overweight or obese. Already at birth, overweight children had a significantly higher mean BMI SDS than normal weight 8-year-olds (p < .001) and this difference increased in consecutive age groups in infancy and childhood. A relatively large increase in BMI during the first 9 months was identified as important for being overweight at 8 years. BMI SDS at birth was associated with overweight at 8 years of age (OR, 1.8; 1.6–2.0), and with obesity (OR, 1.8; 1.4–2.3). The Odds Ratios for the BMI SDS and change in BMI SDS further increased up to 1 year of age became very high from 2 years of age onwards.

**Conclusions:**

A high birth weight and an increasing BMI SDS during the first 9 months and high BMI from 2 years of age proved important landmarks for the onset of being overweight at 8 years of age. The risks of being overweight at 8 years appear to start very early. Interventions to prevent children becoming overweight should not only start at a very early age but also include the prenatal stage.

## Background

Being overweight and obese in childhood have become major challenges for public health as comorbidities start emerging in childhood [1] and many overweight children will develop into obese adults [2,3]. Prevention is considered the optimal strategy, as the treatment of obesity once it develops is notoriously difficult [4]. The identification of important ages for the development of being overweight is an essential aspect of preventive efforts [5,6].

Various age stages in infancy [7-10] and early childhood [11-18] have been associated with the later development of being overweight and obese, but there is currently no consensus on which period is most critical. Although most studies indicate that early weight gain is associated with being overweight later in life, they are often hampered by limitations such as a small number of participants [13,15,17], not being population based [15,18], or being based on self-reported data [11]. Larger population-based studies are needed to evaluate the association of early growth patterns with the subsequent development of being overweight and obese.

The objective of the present population-based study was to compare characteristics of early growth in overweight and obese children with those in non-overweight children to identify important ages for the onset and development of being overweight and obese.

## Methods

### Childhood population

Anthropometric measurements were obtained from a nationally representative sample of 3172 third-grade pupils, with a mean age of 8.3 years (range 7.3–9.6) in the Norwegian Child Growth Study 2010. This study was a part of the European Childhood Obesity Surveillance Initiative [19]. One hundred and twenty-seven schools were selected using a stratified two-stage sampling design [20]. The attendance rate of participants was 89% (1% of the parents refused, and 10% of the third-graders were absent from school on the day of examination).

Data on height and weight of children between birth and 8 years of age were collected from the Medical Birth Registry of Norway and from the health records of previous routine measurements in well-child clinics and school health centers. Records were linked with the unique personal identification number of each child. Routine measurements in Norway are scheduled at birth and at the age of 6 weeks, 3, 6, 9, 12, 15, 18 and 24 months, and 3, 4 and 6 years. Of all children attending the examination, 2920 could be tracked (93%) with an average of nine data points between birth and 8 years of age; 252 could not be tracked or had fewer than three recorded measurements in the period under consideration. Most of these 252 children had been born abroad and had moved to Norway during the preceding years.

### Data analysis

BMI values were converted to SDS using the Norwegian BMI growth reference [21]. For each participant, the BMI SDS at the target ages of 0, 0.25, 0.50, 0.75, 1, 1.50, 2, 4, 6, and 8 years of age was estimated with a piecewise linear mixed effect model with knots at the target ages, and a random effect for each knot. This sequence of linear regressions connecting at target ages, also known as the broken stick model [16], uses data from individuals and from the whole sample to obtain an estimate of the most likely BMI at the target ages of each participant. These estimates were used for further analysis of the BMI at specific target ages, and the change in BMI between target ages, but no assumptions were made about the precise BMI trajectory between target ages. The model was fitted with the lme4 package in R: (R Foundation for Statistical Computing, Vienna, Austria; http://www.r-project.org). The expected value of the BMI SDS at a given age break is referred to as the “status score”, and the change between the status score at the start and the end of an age interval as the “change score”. The BMI at 8 years of age was used to classify subjects as being overweight or not, or as being obese or not, using the age- and sex-specific International Obesity Task Force (IOTF) criteria. These BMI criteria at 8 years of age are 18.44 for being overweight in boys and 18.35 in girls. The corresponding criteria for obesity are 21.60 in boys and 21.57 in girls [22]. According to this, the definition of being overweight includes obesity, unless otherwise specified.

For determining important ages associated with the development of being overweight, we assessed the association between status scores and change scores with the subjects’ overweight and obesity status at 8 years of age using logistic regression, and with two-sample t-tests. In addition, logistic regression with a proportional odds model was used to estimate the combined OR for overweight and obesity (23). Logistic regression was performed for status scores and change scores separately, and for change scores together with the status score at the start of each age interval.

### Ethics

The Regional Committee for Medical Research Ethics approved this study. Written consent for participation in the study, and for retrieval of data from the Medical Birth Registry and from the well-child clinic health records, was obtained from one parent of each participant.

## Results

**Figure 1:**
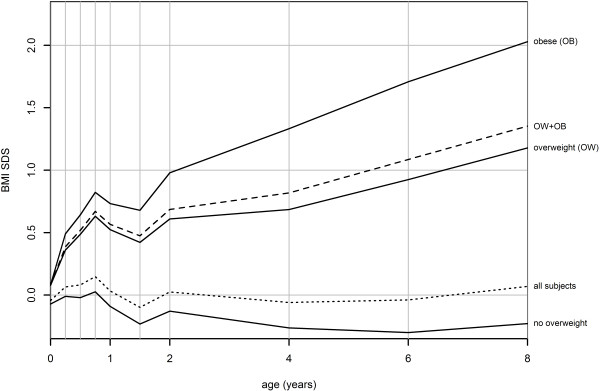
**Trajectories of mean BMI SDS values at target ages according to IOTF BMI status at age 8 years (the “broken stick” model).** The different lines represent the mean BMI SDS in children with no overweight in the total population; those being overweight (but not obese); those being overweight (OW); being overweight and obese (OW + OB) and being obese (OB) at 8 years of age (N = 3172).

The prevalence of being overweight at 8 years of age among all participating children according to the IOTF criteria was 20.4% (range 19.0–21, 8%; N = 647) and that of obesity 4.3% (3.6–5.0%, N = 136). Children who were overweight or obese at 8 years of age had a higher mean BMI SDS than normal weight children at all ages (Table 1, Figure 1). A significant difference in mean BMI SDS of 0.15 between overweight and normal weight children was already present at birth. From birth onwards, the difference in average BMI SDS between these groups increased to 0.54 at 6 months of age, 0.66 at one year, and 1.08 at 4 years of age (all p < .001; Table 1). A similar pattern with even larger differences was observed in obese children although the results were not significant at birth. In addition, the mean gain in BMI between target ages was significantly higher in children who were overweight or obese at 8 years of age compared with children who were not, except between 0.75 and 1.00 years of age. The largest mean rate of change was observed between birth and 9 months of age (Table 2).

**Table 1:** Mean body mass index (BMI) for the total sample and BMI standard deviation score (SDS) status at various target ages in children with and without overweight and obesity at 8 years of age

**Age Years, N**		**Total sample BMI (± SD)**	**Not overweight**^**c, **^**at 8 years of age Mean BMI SDS (95% CI)**	**Overweight**^**c,d **^**at 8 years of age Mean BMI SDS (95% CI)**	***p*-value**^**a,b**^	**Not obese**^**c,e **^**at 8 years of age Mean BMI SDS (95% CI)**	**Obese**^**c **^**at 8 years of age Mean BMI SDS (95% CI)**	***p*-value**^**a,b**^
0.00	2920	14.20 (1.81)	-0.07 (–0.11 - –0.03)	0.08 (0.00 - 0.16)	0.001	-0.05 (–0.08 - –0.01)	0.08 (–0.08 - 0.25)	0.132
0.25	2498	16.63 (1.51)	-0.01 (–0.04 - 0.02)	0.39 (0.32 - 0.45)	0.000	0.05 (0.02 - 0.08)	0.49 (0.35 - 0.63)	0.000
0.50	2514	17.19 (1.51)	-0.02 (–0.05 - 0.01)	0.52 (0.45 - 0.59)	0.000	0.06 (0.03 - 0.09)	0.64 (0.49 - 0.79)	0.000
0.75	2855	17.30 (1.52)	0.03 (–0.01 - 0.06)	0.67 (0.59 - 0.75)	0.000	0.12 (0.09 - 0.15)	0.82 (0.66 - 0.98)	0.000
1.00	2714	17.00 (1.41)	-0.09 (–0.13 - –0.06)	0.57 (0.50 - 0.64)	0.000	0.00 (–0.03 - 0.04)	0.73 (0.58 - 0.89)	0.000
1.50	808	16.39 (1.40)	-0.23 (–0.27 - –0.20)	0.47 (0.40 - 0.55)	0.000	-0.13 (–0.16 - –0.10)	0.68 (0.52 - 0.84)	0.000
2.00	2445	16.40 (1.43)	-0.13 (–0.17 - –0.09)	0.69 (0.61 - 0.76)	0.000	-0.01 (–0.05 - 0.02)	0.98 (0.81 - 1.15)	0.000
4.00	2391	15.80 (1.33)	0.26 (–0.30 - –0.23)	0.82 (0.76 - 0.88)	0.000	-0.12 (–0.15 - –0.08)	1.33 (1.20 - 1.46)	0.000
6.00	2316	15.83 (1.59)	0.30 (–0.33 - –0.27)	1.09 (1.04 - 1.13)	0.000	-0.11 (–0.14 - –0.08)	1.71 (1.63 - 1.79)	0.000
8.00	3172	16.85 (2.40)	-0.23 (–0.26 - –0.20)	1.35 (1.32 - 1.39)	0.000	-0.01 (–0.04 - 0.02)	2.03 (1.97 - 2.09)	0.000

SD, standard deviation, CI, confidence interval.

^a^Two-sample independent *t*-test.

^b^Statistically significant (p < .001).

^c^Broken stick model (N = 3172).

^d^Overweight includes obesity.

^e^The “not obese” group includes all other children, also those that are overweight.

**Table 2:** **Absolute changes in BMI SDS and rates of change in BMI SDS during each period in children who were or were not overweight or obese at 8 years of age**^**c**^

**Age, years**	**Not overweight at 8 years**^**a,d**^	**Overweight**^**d **^**at 8 years**^**d**^	**Obese at 8 years**^**a**^	**Overweight**^**d **^**vs. not overweight**		**Obese versus not obese**^**e**^	
				**ΔSDS**^**b **^**(95% CI)**	**ΔSDS/year**^**b **^**(95% CI)**	**ΔSDS**^**b**^**(95% CI)**	**ΔSDS/year**^**b **^**(95% CI)**
0.00–0.25	0.06	0.31	0.41	0.25 (0.16–0.34)*	0.99 (0.63–1.35)	0.31 (0.14–0.49)*	1.25 (0.55–1.95)
0.25–0.50	-0.01	0.13	0.15	0.14 (0.11–0.17)*	0.57 (0.44–0.69)	0.14 (0.08–0.21)*	0.57 (0.31–0.82)
0.50–0.75	0.05	0.15	0.18	0.11 (0.09–0.12)*	0.42 (0.35–0.50)	0.12 (0.08–0.16)*	0.47 (0.32–0.63)
0.75–1.00	-0.12	-0.10	-0.09	0.01 (0.0–0.03)	0.05 (–0.01–0.11)	0.03 (0.0–0.05)	0.10 (0.01–0.22)
1.00–1.50	-0.14	-0.09	-0.05	0.05 (0.03–0.07)*	0.10 (0.05–0.14)	0.08 (0.03–0.13)*	0.16 (0.07–0.25)
1.50–2.00	0.10	0.21	0.30	0.11 (0.08–0.14)*	0.22 (0.16–0.27)	0.18 (0.12–0.25)*	0.37 (0.24–0.50)
2.00–4.00	-0.13	0.13	0.35	0.27 (0.22–0.31)*	0.13 (0.11–0.15)	0.45 (0.36–0.55)*	0.23 (0.18–.028)
4.00–6.00	-0.04	0.27	0.38	0.30 (0.27–0.34)*	0.15 (0.14–0.17)	0.37 (0.30–0.44)*	0.18 (0.15–0.22)
6.00–8.00	0.07	0.27	0.32	0.20 (0.17–0.22)*	0.10 (0.09–0.11)	0.22 (0.18–0.27)*	0.11 (0.09–0.13)

CI, confidence interval.

^a^Change in BMI SDS (absolute change over the specified interval).

^b^Difference between groups of the absolute change over the specified interval (ΔSDS) of the change expressed as ΔSDS/year.

^c^Broken stick model (N = 3172).

^d^Overweight includes obesity.

^e^The “not obese” group includes all other children, also those that are overweight.

T-tests were used for comparing groups: *p < .001.

The OR for being overweight or obese at 8 years of age (per unit increase in BMI SDS at the target ages) increased with age, first gradually, and more rapidly from 2 years onward (Table 3). The OR values were statistically significant at all ages, and increased to very high numbers by the age of 4 years. The BMI SDS status at age 4 was thus highly predictive—almost deterministic—for being overweight or obese at age 8. When the change scores were corrected for the status score at the start of the interval, the results obtained were largely similar to the uncorrected change scores, except from 0.75 – 1.00 year and from 2 years of age onwards, when they become much larger. From two years of age onwards, a child with a high and still increasing BMI SDS was almost certainly overweight or obese by the age of 8 years.

**Table 3:** **Odds ratios and 95% CI for being overweight or obese at 8 years of age according to the BMI SDS at each target age (status) and change in BMI SDS during each period (change)**^**a**^

	**Overweight**			**Obese**		
**Age, years Age intervals**	**Status**^**b **^**OR (95% CI) for being overweight at 8 years per 1 SDS score increase at the target age**	**Change**^**c **^**OR (95% CI) for being overweight at 8 years per 1 SDS score increase between target ages**	**Change and status**^**d **^**OR (95% CI)**	**Status**^**b **^**OR (95% CI) for being obese at 8 years per 1 SDS score increase at target age**	**Change**^**c **^**OR (95% CI) for being obese at 8 years per 1 SDS score increase between target ages**	**Change and status**^**d **^**OR (95 % CI)**
0.00	1.8 (1.6–2.0)		1.8 (1.7–2.0)	1.9 (1.5–2.3)		1.8 (1.4–2.3)
0.00–0.25		1.2 (1.1–1.4)	1.8 (1.6–2.0)		1.3 (1.1–1.5)	1.9 (1.5–2.4)
0.25	2.0 (1.8–2.3)		1.9 (1.7–2.1)	2.1 (1.7–2.5)		1.9 (1.5–2.4)
0.25–0.50		3.1 (2.4–4.0)	3.6 (2.7–4.7)		2.9 (1.8–4.7)	3.4 (2.0–5.6)
0.50	2.1 (1.9–2.4)		1.9 (1.7–2.1)	2.2 (1.8–2.6)		1.9 (1.6 –2.4)
0.50–0.75		8.5 (5.7–13.0)	6.6 (4.3–10.0)		9.4 (4.4–19.9)	7.2 (3.2–10.69)
0.75	2.4 (2.1–2.7)		2.5 (2.3–2.9)	2.5 (2.0–3.0)		2.7 (2.1–3.3)
0.75–1.00		1.6 (0.9–2.6)	10.1 (6.0–11.0)		2.4 (0.8–6.6)	16.4 (5.4–40.9)
1.00	2.5 (2.3 – 2.8)		2.5 (2.2–2.8)	2.7 (2.2–3.4)		2.6 (2.1–3.3)
1.00–1.50		2.2 (1.5 3.2)	3.4 (2.3–5.1)		3.6 (1.8––7.4)	5.7 (2.7–10.2)
1.50	2.7 (2.4 – 3.0)		2.6 (2.3–2.9)	3.1 (2.5–3.9)		2.9 (2.3–3.6)
1.50–2.00		2.6 (2.0–3.5)	3.2 (2.4–4.3)		5.0 (3.0–8.6)	6.57 (3.7–10.2)
2.00	5.8 (5.0–6.8)		6.2 (5.3–7.4)	9.4 (7.0–13.0)		10.2 (7.4–14.6)
2.00–4.00		3.4 (2.8–4.5)	20.7 (19.3–30.7)		6.0 (4.2–8.5)	72.7 (40.0–100.4)
4.00	63.8 (45.5–91.5)		> 100	> 100 (90.9– > 100)		>100
4.00–6.00		12.5 (9.4–16.8)	>100		11.5 (7.3–18.1)	>100
6.00	>100		>100	>100		>100
6.00–8.00		25.7 (17.4–38.3)	>100		23.2 (12.0–45.1)	>100

CI, confidence interval.

^a^Broken stick model (N = 3172).

^b^Status: the risk of being overweight according to the BMI at that age.

^c^Change: the risk of being overweight according to the BMI change between the status score at the start and the end of an age interval.

^d^Change-corrected for status: e.g., status at 1 + change from 1 to 1.5.

## Discussion

In the current population-based longitudinal study, being overweight or obese in 8-year-old children was associated with a higher mean BMI throughout infancy and childhood, and was already recognizable at birth. The first 9 months of life showed a marked increase in mean BMI in overweight and obese children, with a further separation from children of normal weight after 2 years of age. From 2 years and onwards, the ORs increased steeply, showing a strong association between a high or increasing BMI and being overweight or obese at 8 years of age.

The prevalence of being overweight at 8 years of age in this sample was 20.4%, similar to data from the Bergen Growth Study [23]. According to the Norwegian Child Growth Study [24], the prevalence of overweight 8-year-old children in Norway increased between 2008 and 2010, but in 2012 this seems to have reached a plateau, as reported in some other countries [25-27].

We found that a higher birth weight predicted being overweight at the age of 8 years, suggesting that prenatal development affects later childhood growth. Similar findings have also been reported by Hui et al. [9] and reviewed by Rogers [28]. Thus, there seems to be an association between birth weight, subsequent BMI and the risk of becoming overweight in children and young adults. Eriksson et al. [29] found that a higher maternal BMI during pregnancy was associated with more rapid growth in the offspring and an increased risk of becoming obese in adulthood. Findings from a large cohort study of the impact of the intrauterine environment on later childhood adiposity suggested that the association between the BMI of the mother and her offspring could be explained more by shared familial risk factors rather than the intrauterine environment [30]. Whether this association is caused by intrauterine programming, by genetic factors, or is mainly a result of lifestyle factors operating in postnatal life has not yet been established [31].

The current data show that a rapid increase in the BMI during the first year of life, and especially between the ages of 6 and 9 months, significantly increases the risk of being overweight at 8 years of age (Table 3). Other authors have found similar results when studying weight gain in early childhood [13,14] and weight gain during adolescence [7,8,10].

In the current study, 8-year-old overweight children had an average change score between 9 and 12 months of age that was similar to that in normal weight children (Table 2). However, logistic regression showed that the risk of becoming overweight was significantly larger when the BMI increased particularly during this period (Table 3, change scores corrected for status). This apparently contradictory result possibly arose from the distribution of change scores: i.e., the majority of overweight subjects had a change score that was similar to that in normal weight subjects, but a relative increase in BMI SDS still involves a higher risk of becoming overweight.

In the current study, children who were overweight at 8 years of age had already shown more rapid gains in their BMI at than normal weight children almost all ages of growth up to that age. Although other studies have also highlighted the importance of early growth for the later development of being overweight and obese, the suggested critical time points have differed. Thus, de Kroon et al. [16] did not find that rapid growth during the first years of life was a predictor for being overweight at the age of 18. In contrast, Stettler et al. suggested the importance of the first week of life for becoming overweight at 20–34 years of age in formula-fed babies [32]. Péneau et al. [12] found that rapid growth during infancy and being large at 12 months of age was associated with being overweight at 7–9 years of age. Lagström et al. [17] found that overweight children at 13 years of age had gained more weight than their normal-weight peers from the age of 2 or 3 years onward. Although our study showed that a rapid gain in BMI was a risk factor for being overweight at 8 years, we cannot confirm that this is the case for those who are overweight as adults.

The relative risk of being overweight at 8 years increased with age according to the increase in BMI at specific target ages. In our study, we found that the OR for being overweight at 8 years of age increased from 2 years and onward. Thus, having a high BMI at age 6 years had an extremely high association with being overweight at age 8. Botton et al. [11] described two periods in early childhood that were associated with later risk of being overweight or obese as an adolescent: up to 6 months and from 2 years onward. In a recent study among overweight children aged 10–12 years, Harrington et al. [15] reported that more than half of the children had already been overweight at 2 years of age, and defined the “tipping point” of weight development to be 22 months of age. Similarly, De Kroon et al. [16] found the age between 2 and 6 years to be the most important growth period for predicting adult overweight.

The main strength of the present study was its population-based design with a high participation rate and measurements of weight and height performed longitudinally at well-child clinics/school health services. The average of nine measurements taken between birth and 8 years of age offered opportunities to study the potential of previous measurements to predict whether children would be overweight at age 8. The limitations of the study were some variation in the children’s age at the time of measurements, and missing data. These shortfalls were addressed by applying the ‘;broken stick’ method, which allows obtaining an estimate of the BMI for each individual at the exact target age, but which might also give estimates that are closer to the mean when data are missing. This implies that any tests of differences will be conservative, and might possibly underestimate the effects of BMI changes during periods in which fewer measurements are recorded [16]. A selection bias with regard to ethnicity cannot be excluded, as the majority of the children whose data could not be retrieved or who had fewer than three recorded measurements in the period were born abroad and had moved to Norway during the preceding years.

## Conclusions

The results of this study suggest that a high birth weight and an increasing BMI are throughout infancy and childhood associated with being overweight at 8 years of age. Routine assessments of height and weight as well as recognition of excessive weight gain are essential to prevent the onset of being overweight in children, as parents do not always recognize their child as having a weight problem [33,34]. There is also a need for the development of universal preventive programs and resources in primary health care to deal with this public health challenge.

## Abbreviations

BMI: Body mass index; CI: Confidence interval; IOTF: International obesity task force; OR: Odds ratio; SDS: Standard deviation score.

## Competing interests

The authors have no conflicts of interest to disclose. No external funding was secured for this study. The authors have no financial relationships to disclose relevant to this article.

## Authors’ contributions

KG conceptualized and designed the study, contributed to data collection and drafted the initial manuscript. MR carried out the initial analyses, reviewed and revised the manuscript. BHS provided statistical support and reviewed the manuscript. PBJ contributed to professional support, and reviewed and revised the manuscript. KKL contributed to data collection, and reviewed the manuscript. SH contributed to data collection, and reviewed the manuscript. RH conceptualized and designed the study, coordinated and supervised data collection, and reviewed the manuscript. All authors have approved the final manuscript as submitted.

## Pre-publication history

The pre-publication history for this paper can be accessed here:

http://www.biomedcentral.com/1471-2458/14/160/prepub
